# Clinical and genetic analysis of a case with centronuclear myopathy caused by SPEG gene mutation: a case report and literature review

**DOI:** 10.1186/s12887-021-02656-6

**Published:** 2021-04-29

**Authors:** Gang Zhang, Min Xu, Tingting Huang, Wenxin Lin, Jinglin Chen, Wangyang Chen, Xingzhi Chang

**Affiliations:** 1 Department of Neurology, Children’s Hospital of Nanjing Medical University, Nanjing, China; 2 Kaiumph Medical Diagnostics Co, Ltd, Beijing, 100102 China; 3 Department of Pediatrics, Peking University First Hospital, No.1 Xi’an Men Street, West District, Beijing, 100034 China

**Keywords:** Centronuclear myopathy, *SPEG* gene, Clinical phenotype, Electron microscopy study, Histochemical staining

## Abstract

**Background:**

Centronuclear myopathy (CNM), a subtype of congenital myopathy (CM), is a group of clinical and genetically heterogeneous muscle disorders. Since the discovery of the SPEG gene and disease-causing variants, only a few additional patients have been reported.

**Case presentation:**

The child, a 13-year-old female, had delayed motor development since childhood, weakness of both lower extremities for 10 years, gait swinging, and a positive Gower sign. Her distal muscle strength of both lower extremities was grade IV. The electromyography showed myogenic damage and electromyographic changes. Her 11-year-old sister had a similar muscle weakness phenotype. Gene sequencing revealed that both sisters had SPEG compound heterozygous mutations, and the mutation sites were c.3715 + 4C > T and c.3588delC, which were derived from their parents. These variant sites have not been reported before. The muscle biopsy showed the nucleic (> 20% of fibers) were located in the center of the cell, the average diameter of type I myofibers was slightly smaller than that of type II myofibers, and the pathology of type I myofibers was dominant, which agreed with the pathological changes of centronuclear myopathy.

**Conclusions:**

The clinical phenotypes of CNM patients caused by mutations at different sites of the SPEG gene are also different. In this case, there was no cardiomyopathy. This study expanded the number of CNM cases and the mutation spectrum of the SPEG gene to provide references for prenatal diagnosis and genetic counseling.

## Background

Centronuclear myopathies (CNM) are a group of congenital myopathies with clinical and genetic heterogeneity. Their names are derived from increased numbers of central nuclei within myofibers and type I myofiber atrophy and pathological predominance on muscle biopsies [1]. The disease was first reported by Spiro et al. in 1966 [2]. The clinical manifestations often include hypotonia, limb weakness, drooping eyelids, extraocular muscle paralysis, and a slowly progressive clinical course. The pathogenic gene has been identified in 60–80% of CNM patients, and a new pathogenic gene, the striated preferentially expressed gene (*SPEG*), has been discovered in recent years. There are only a few related reports caused by *SPEG* mutation at present. In our study, we found the two mutations in *SPEG* and neither of these two sites has been reported before. This study reported the data of a child with CNM caused by SPEG gene mutation, to improve clinicians’ understanding of the disease and to expand the gene spectrum of SPEG to provide help for early diagnosis and genetic counseling of such patients.

## Case presentation

**Fig. 1:**
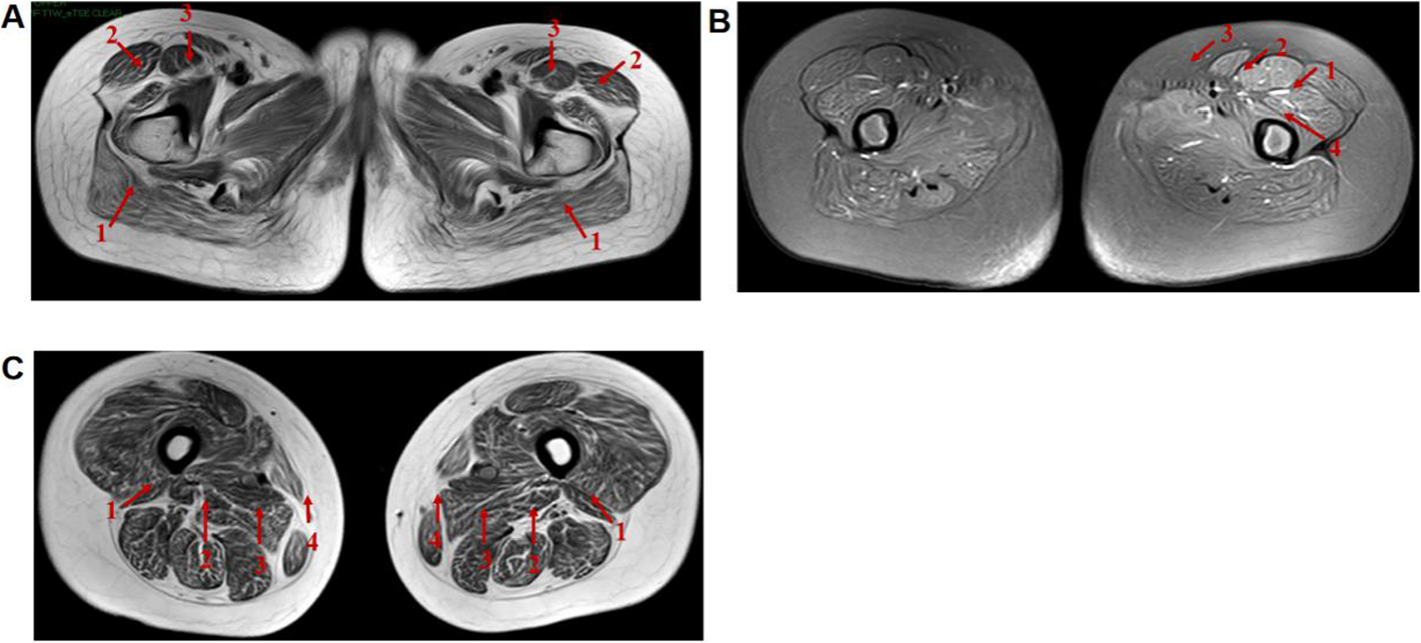
**a** T2WIF transverse position shows the fat infiltration of the bilateral gluteus maximus (arrow 1), tensor fascia lata (arrow 2), and rectus femoris (arrow 3). **b** T2WI fat suppression transverse position shows swelling of the bilateral lateral femoris (arrow 1), rectus femoris (arrow 2), sartorius (arrow 3), and midfemoris muscle (arrow 4), intermuscular edema, and the obvious left side. **c** T2WI transection position shows the fat infiltration of the bilateral biceps femoris (arrow 1), semitendinosus (arrow 2), semimembranosus (arrow 3) and gracilis (arrow 4), and increased intermuscular fat

The patient is a 13-year-old female who visited the clinic for the weakness of both lower limbs for 10 years. More than 10 years ago, the child developed weakness in both lower extremities, easy fatigue, unstable gait, easy falling when walking, difficulty standing up after falling or squatting, and difficulty climbing stairs, and she was unable to run and jump. These conditions were not obvious to the family at the beginning of the illness, and her condition gradually worsened. She suffered from delayed motor development since childhood. She attained head control at 3 months, moved into a sitting position at 10 months, and walked at 2 years. Her intellectual development was normal before and after the onset of the disease. She was G5P2, delivered naturally at term, and there were no abnormalities during pregnancy or the perinatal period. Her parents were in good health and denied any consanguinity. The 11-year-old sister had similar muscle weakness since childhood. The patient had clear consciousness, good orientation, verbal expression ability and memory. Her neck was soft, cardiopulmonary and abdominal examinations were normal, distal muscle strength of both upper extremities was grade V, proximal muscle strength of both upper extremities was grade IV, distal muscle strength of both lower extremities was grade V, and proximal muscle strength of both lower extremities was grade IV. She had normal muscle tension of the extremities, normal temperature and pain, positive Gower sign, slightly unstable gait, stable finger-nose test, and stable heel-knee-shin test. Romberg sign was negative, the bilateral knee reflex was not elicited, and pathological reflex was negative. Her serum creatine kinase level was normal. EMG examination revealed myogenic damage changes, accompanied by mild neurogenic damage changes. Echocardiography indicated no abnormality. EKG revealed sinus tachycardia, occasional ventricular premature contraction, occasional atrial premature contraction and ST-segment changes. A plain MRI scan of the bilateral thigh muscles revealed that both hips and thigh muscles had fatty infiltration with mild edema (Fig. 1a, b, c).

**Fig. 2:**
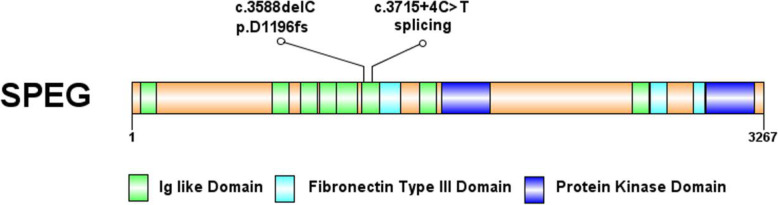
Distribution of alterations across the schematic of SPEG. Domains are also indicated

**Fig. 3:**
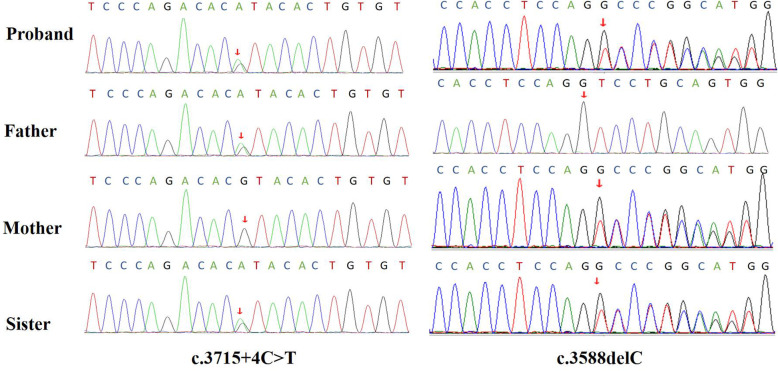
Two mutations in exon 13 of the SPEG gene in the child: c.3715 + 4C > T splicing mutation, derived from the father; c.3588delC frameshift mutation, derived from the mother; her younger sister carries the same mutation site

Gene sequencing revealed that the child had SPEG gene mutations, and the mutation sites were c.3715 + 4C > T and c.3588delC, which are located on exon 13. The two mutations identified in our case are marked in the SPEG schematic (Fig. 2). Neither of these two sites has been reported so far. Further family genealogical verification revealed that her sister carried two identical mutation sites, c.37 15 + 4C > T and c.3588delC from the father and mother, respectively (Fig. 3). Both parents were heterozygous carriers, and the child had a compound heterozygous mutation, which conforms to the law of autosomal recessive inheritance. Among them, c3588dL was rated as suspected pathogenic variation by ACMG, while 371544CT was rated as clinically unknown. Through the prediction of Mutation Taster, it was found that the mutation of c.3588delC led to a change in the amino acid sequence. Compared with wild type, expression of the amino acid sequence was significantly reduced, and the corresponding protein structure prediction was found based on Mutation Taster analysis.

**Fig. 4:**
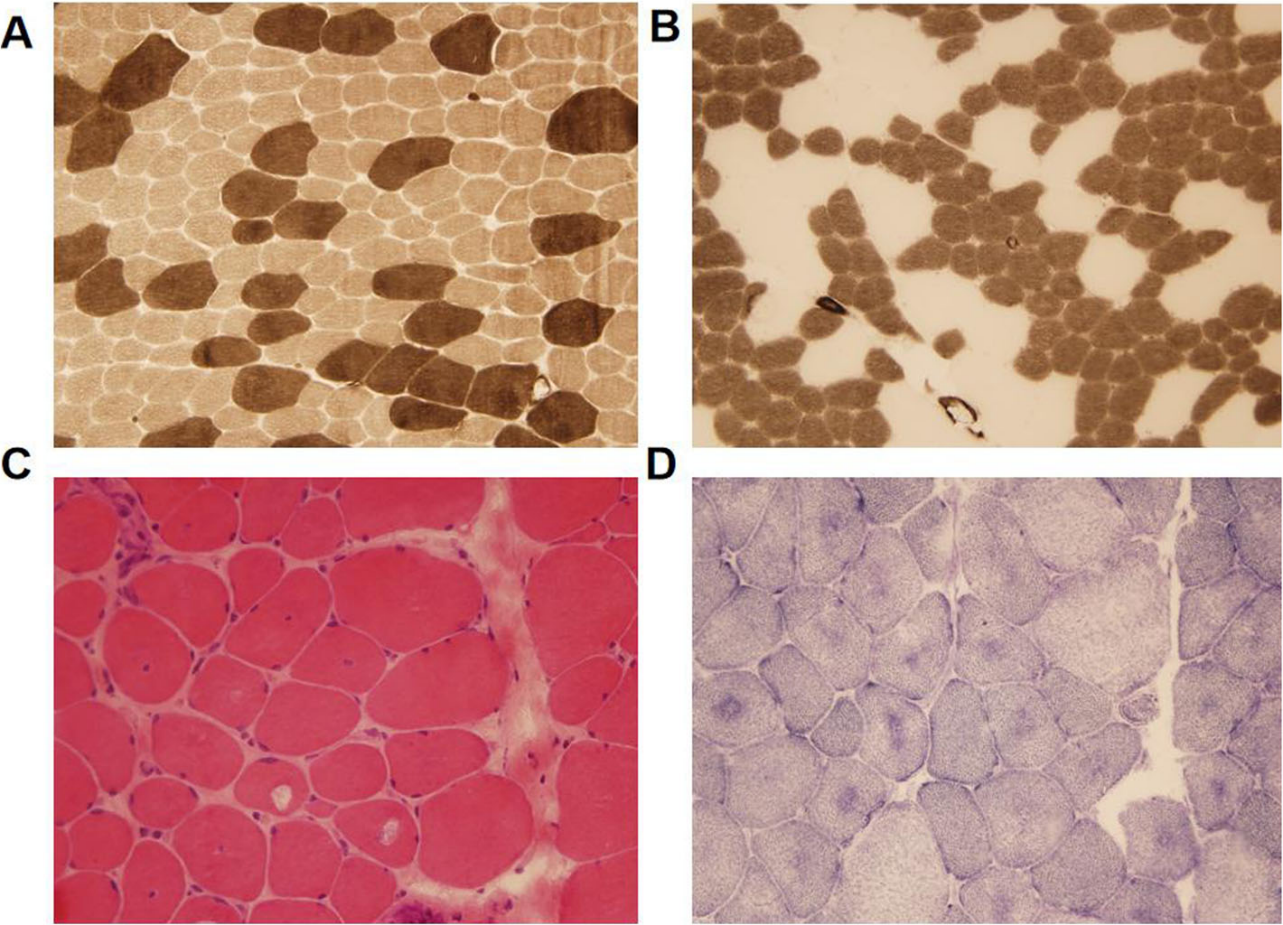
Histological examination of the patient’s muscle biopsies: **a** The acid reaction series of ATPase showed checkerboard distribution of myofibers, and the pathology of type I myofibers was dominant. There are two types of atrophic myofibers, mainly type I. The diameter of type I myofiber is generally smaller than that of type II myofiber. **b** The alkaline reaction series of ATPase confirmed the abovementioned distribution characteristics of myofibers. Slightly atrophied-type II fiber is 15–25 μm in diameter. **c** HE staining shows that the nuclei of some myofibers are located in the center of the cell. **d** NADH1 staining showed that the center of some myofibers was slightly darker

The child had a biceps muscle biopsy. The main pathological change showed the nucleic (> 20% of fibers) were located in the center of the cell, the average diameter of type I myofibers was slightly smaller than that of type II myofibers, and the pathology of type I myofibers was dominant. There was no inflammatory cell infiltration, myofiber necrosis, or other characteristic structural change in the myofibers, and these analyses agree with the pathological changes of centronuclear myopathy (Fig. 4). There was no histologic evidence of inflammatory myopathy, myodystrophy, or fat or glycogen accumulative myopathy.

## Discussion and conclusions

CNM can be divided into the following categories: 1) X-linked recessive hereditary myotubular myopathy: its pathogenic gene is MTM1, which encodes the MTM1 protein. This form is characterized clinically by male-onset and is the most common severe subtype of CNM. Most affected males die from respiratory failure before 1 year of age; however, a very small number of affected males can survive childhood or even adolescence. Female MTM1 heterozygous carriers can also develop the disease, which may be related to thr nonrandom inactivation of the X chromosome. 2) Autosomal dominant genetic type: pathogenic genes of this type are DNM2, CCDC78, BIN1, and MTMR 14. The range of clinical manifestations is wide, with varying severity. 3) Autosomal recessive genetic type: disease-causing genes are RYR1, TTN, and SPEG, and patients with this type have moderate to severe clinical phenotypes [3–9]. In 2014, Agrawal PB et al. reported 3 cases of SPEG gene mutation in patients for the first time and determined that SPEG gene mutation can cause CNM [10], and the related diseases are all CNM. Thus far, there have been 11 SPEG-related cases reported, and the related diseases are all CNM.

The SPEG gene is located on chromosome 2q35, contains 51 exons, and encodes striated muscle preferentially expressed protein kinase (SPEG), which is essential for the formation of the muscle cell skeleton. Current studies suggest that SPEG, as the molecular partner of MTM1, interacts with MTM1 as its main pathogenic mechanism. The interaction between the two causes Ca2+ homeostasis disorder in the sarcoplasmic reticulum of skeletal muscle and the myocardium and affects the positioning of the nucleus during skeletal muscle maturation, eventually leading to abnormal muscle cell skeleton development. Besides, the four SPEG subtypes that were identified in murine models include SPEGα, SPEGβ, APEG-1, and BPEG, among which SPEGα and SPEGβ are highly expressed in skeletal and cardiac muscle, APEG-1 is highly expressed in vascular tissue, and BPEG is expressed in the brain and aorta. The differences in the expression of SPEG subtypes in different tissues play an important role in the study of patient clinical phenotypes [11–13].

Using “SPEG gene, centronuclear myopathy” in both Chinese and English as keywords, the literature was searched to September 2020 in the following databases, the Chinese Journal Full-text Database (CNKI), Wanfang Data Knowledge Service Platform, the National Center for Biotechnology (NCBI) and biomedical literature database (PubMed), and a total of 6 related reports were retrieved (all in the English literature). The reported SPEG gene variants are all related to CNM. A total of 16 variant sites have been reported including the two sites we discovered in our study as shown in Table 1 [10, 14–18].

**Table 1:** The SPEG variant sites reported so far with references

Patient	Sex	***SPEG*** variant	Reference	Comment
1	F	c.6697C > T;	Agrawal 2014 [10]	p.Gln2233*
2	F	c.4276C > T; c.3709_3715 + 29del36;	Agrawal 2014 [10]	p.Arg1426* p.Thr1237fs
3	F	c.2915_2916delCCinsA; c.8270G > T;	Agrawal 2014 [10]	p.Ala972fs p.Gly2757Val
4	M	c.1627-1628insA;	Wang 2017 [14]	p.Thr544fs
5	M	c.9586C > T;	Wang 2017 [14]	p.Arg3196*
6	M	c.7119C > A;	Wang 2018 [15]	p.Tyr2373*
7	F	c.1071_1074dup; c.4399C > T;	Lornage 2018 [16]	p.Lys359fs p.Arg1467*
8	M	c.9185_9187delTGG;	Qualls 2019 [17]	p.Val3062del
9	F	c.2183delT; c.8962_8963ins25;	Qualls 2019 [17]	p.Leu728fs p.Val2997fs
10	F	c.8710A > G; p.	Tang 2019 [18]	Thr2904Ala
11	F	c.3715 + 4C > T; c.3588delC;	This study	p.D1196fs

* represents termination password

**Fig. 5:**
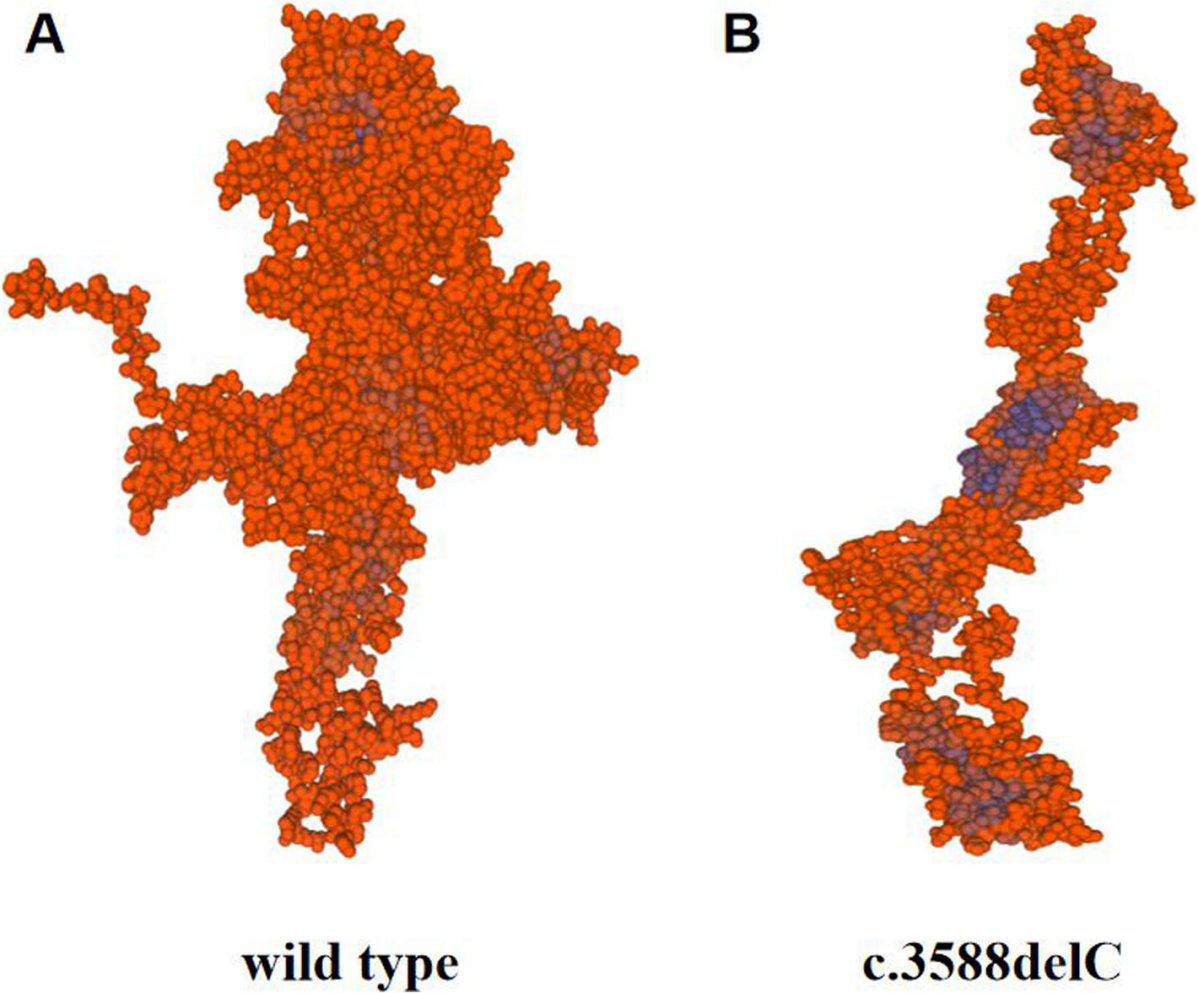
The mutation of the SPEG gene; c.3588delC was predicted by Mutation Taster to cause multiple Ig-like and protein kinase domains to be lost. The protein structure was predicted that **a** was wild type and **b** was mutant

**Fig. 6:**
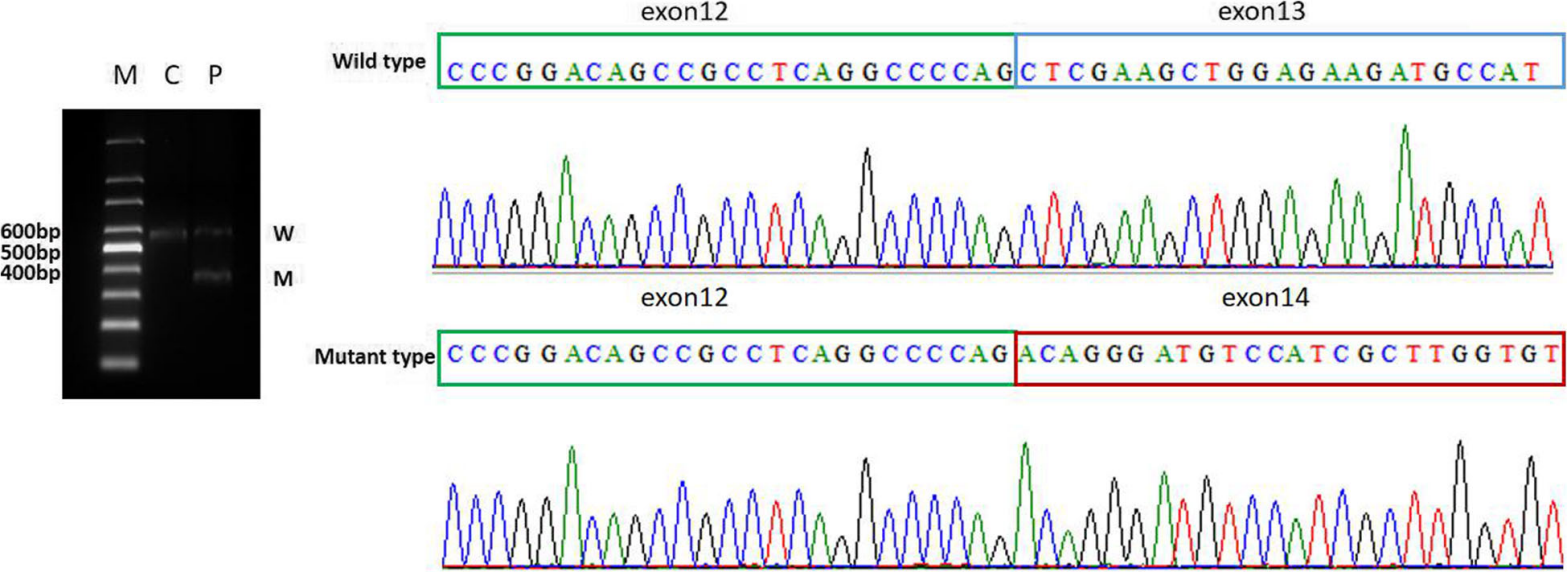
The result of cDNA PCR gel electrophoresis showed that the novel c.3715 + 4C > T splicing mutation produced an abnormal transcript; The PCR product of normal transcript was 608 bp, and that of abnormal transcript was 384 bp. SPEG cDNA forward primer: 5′-GAGCTGCCCGTTTCGACTGCAA-3′. SPEG cDNA reverse primer: 5′-GACCACATCTGTGACATACAGG-3′

The mutation sites c.3588delC and c.3715 + 4C > T found in our study have not been reported before. The pathogenicity prediction c.3588delC sites suggested the c.3588delC site mutation was a pathogenic mutation. Compared with the wild type, the morphology and size of the mutant SPEG protein were significantly changed, which could lead to the loss of multiple Ig-like and protein kinase domains (Fig. 5). While c. 3715 + 4C > T was a splicing site mutation. This mutation destroyed the original splicing site, caused a change in the reading frame, and then resulted in the transcription of abnormal mRNA and the translation of abnormal proteins, which might result in loss of site activity (Table 2). To further prove the pathogenicity of the site, total RNA was extracted from the peripheral blood samples of the patient using TRIzol Reagent (Invitrogen, Carlsbad, CA). Then, the extracted RNA was reverse transcribed to cDNA. cDNA primers were designed upstream and downstream of SPEG gene c.3715 + 4C > T variation. Subsequently, PCR was performed and PCR products were separated. Finally, different size fragment bands were sequencing by ABI 3500. The result of cDNA PCR gel electrophoresis showed that the novel c.3715 + 4C > T splicing mutation produced an abnormal transcript (The PCR product of normal transcript was 608 bp, and that of the abnormal transcript was 384 bp). The abnormal transcript was recovered and followed by Sanger sequencing. Compared with the reference sequence of SPEG gene transcript NM_005876, the c.3715 + 4C > T mutation led to a skipping of exon 13, a non-triple 224 bp size. The absence resulted in a premature termination codon which might be degraded by NMD and lead to loss of function (Fig. 6).

**Table 2:** SPEG gene c.3715 + 4C > T splicing mutation site, predicted by MutationTaster, will lead to the possible inactivation of the donor site (score reduced from 0.73 to 0.35)

Effect	gDNA position	Score	Wt detection sequence	Exon-intron border
Donor decreased	34,533	Wt:0.73/mu:0.35	Wt:ACACAGTGTACGTGT mu: ACACAGTGTATGTGT	ACAG|tgta

Among the 11 SPEG-associated CNM patients reported thus far, there were 4 males and 7 females, of who 2 were a pair of fraternal twin sisters from China. The onset in these cases was all in the neonatal or infantile periods, with 4 cases dying within 3 weeks after birth and 1 case dying at 19 weeks. Their clinical symptoms were no different from that of general CNM patients and were commonly manifested as hypotonia (11/11), delayed motor milestones (7/11), muscle weakness or atrophy (11/11), weakness in sucking or chewing (7/11), ophthalmoplegia (5/11), blepharoptosis (2/11) and postpartum mechanical ventilation support in patients with severe respiratory muscle involvement (2/11). Physical examination revealed malformations, such as high palatine arch (7/11), scoliosis (3/11), joint contracture (2/11), flat or arched feet (2/11), pectus excavatum (1/11) and posterior mandible contraction (1/11). Besides, cardiac involvement was seen in most reported cases (8/11), most of which presented with dilated cardiomyopathy (6/11), followed by mitral or tricuspid valve insufficiency (4/11). In addition to the above common clinical manifestations, a 10-year-old patient reported by Lorange X in 2018 did not develop cardiomyopathy [16], and a patient-reported by Wang H also developed axon neuropathy and myocardial densification insufficiency in addition to CNM symptoms [15]. The child in this study was 13 years old, and the cardiac B-ultrasound showed no abnormalities, which further proved that the CNM caused by the SPEG gene was not accompanied by cardiomyopathy; at the same time, the electromyogram (EMG) of the child also showed mild neurogenic damage of the common peroneal nerve, which was consistent with Wang H’s report. The occurrence of cardiomyopathy may be related to the different mutation sites of the SPEG gene, or it may be caused by the difference in the expression of different subtypes of SPEG in skeletal muscle and the myocardium. In general, because of the small number of reported cases at present, the genotype-phenotype correlation is still unclear. As the number of reported cases accumulates, there may be discoveries in the future. Besides, 9 of 11 patients underwent muscle biopsies, and their common pathological characteristics showed an increase in the proportion of myofibers with central nuclei and predominance of type I myofiber dysplasia. In some patients, the phenomenon of a perinuclear hollow halo of myofibers could be observed, which was consistent with the existing pathological findings.

At present, the treatment of CNM is not unique, and possible treatment options at the cellular and molecular and genetic levels are under study [19]. At present, symptomatic treatment, such as feeding support for chewing weakness, rehabilitation training for limb weakness, drug treatment for heart involvement, and surgery for joint contracture or scoliosis, is most often recommended [20]. For children with a clear diagnosis, multidisciplinary joint long-term management is needed to improve their quality of life. This study expanded the number of CNM cases and the mutation spectrum of the SPEG gene to provide references for prenatal diagnosis and genetic counseling.

## Data Availability

The datasets used and/or analyzed during the current study are available from the corresponding author on reasonable request.

## Abbreviations

CNM: Centronuclear Myopathy
CM: Congenital Myopathy
SPEG: Striated muscle preferentially-expressed Gene
